# An investigation of kindergarten educator reported barriers and concerns and school neighbourhood composition in Ontario, Canada.

**DOI:** 10.23889/ijpds.v7i3.1839

**Published:** 2022-08-25

**Authors:** Natalie Spadafora, Caroline Reid-Westoby, Molly Pottruff, Jade Wang, Eric Duku, Magdalena Janus

**Affiliations:** 1 McMaster University

## Objectives

The COVID-19 pandemic has not impacted everyone equitably, including children (e.g., Li et al., 2021). The objective of this study was to explore the association between school neighbourhood composition and kindergarten educator-reported barriers and concerns regarding children’s learning during the first wave of COVID-19 related school closures in Ontario, Canada.

## Approach

In the spring of 2020, we collected data from Ontario kindergarten educators in an online survey on their experiences and challenges with online learning during the first round of school closures. We asked educators whether they experienced a number of barriers to learning and concerns about returning to school in the Fall. We linked the educator responses to 2016 Canadian Census variables based on the school postal code. Poisson regression analyses were used to determine if there was an association between neighbourhood composition and the number of barriers and concerns reported by kindergarten educators.

## Results

Educators (n = 2569; 74.2% kindergarten teachers, 25.8% early childhood educators; 97.6% female) who taught at schools in neighbourhoods with lower median income reported a greater number of barriers to online learning (e.g., students' lack of access to electronic devices) and concerns regarding the return to school in the fall of 2020 (e.g., concerned about differences in how much students learned during the school closures). Educators also reported a greater number of concerns regarding the return to the classroom in neighbourhoods with a greater proportion of single-parent families.

## Conclusion

Our study confirms that the educational impacts of the COVID-19 pandemic may not have been felt equitably even by kindergarten children, as educators teaching in schools in lower SES neighbourhoods reported both more barriers to online learning, and more concerns about returning to the classroom in September 2020.

